# Evaluation of Serum Cortisol Levels and Response to Cosyntropin Test in Methadone-treated Opium Addicts

**DOI:** 10.5812/aapm-135206

**Published:** 2023-06-03

**Authors:** Faegheh Zojaji, Alireza Khalesi, Afsane Bahrami, Seyed Ali Ebrahimi, Mahmoud Ganjifard

**Affiliations:** 1 Birjand University of Medical Sciences, Birjand, Iran; 2 Clinical Research Development Unit of Akbar Hospital, Mashhad University of Medical Sciences, Mashhad, Iran; 3 Student Research Committee, Mashhad Islamic Azad University of Medical Sciences, Mashhad, Iran; 4 Department of Anesthesiology and Critical Care, Birjand University of Medical Sciences, Birjand, Iran

**Keywords:** Adrenal Insufficiency, Cortisol, Cosyntropin Test, Methadone, Opioids

## Abstract

**Background:**

Opium has been used for thousands of years for medical and analgesic purposes, and its misuse has also increased in recent years. Methadone, a synthetic opioid, has been used as an analgesic and to help patients quit opium addiction. However, some evidence suggests that long-term use of opioids can affect the hypothalamic-pituitary-adrenal axis.

**Objectives:**

We aimed to evaluate the serum cortisol level and response to the cosyntropin stimulation test in opium addicts on methadone treatment.

**Methods:**

The study was conducted in November 2019 at Imam Reza Hospital Rehab Center, Birjand, Iran. Thirty-eight methadone-treated opium addicts participated in the study. A blood sample was initially obtained, then 250 µg intramuscular cosyntropin was injected. After 30 and 60 minutes, two other blood samples were obtained. The data were analyzed using SPSS.

**Results:**

There was a significant difference between serum cortisol levels and the normal value in methadone users (9.46 ± 5.42 vs. 14 µg/dL) (P < 0.001). The mean response to the cosyntropin stimulation test in methadone users was 9.34 ± 8.11 µg/dL. Also, 55% of the participants had adrenal insufficiency.

**Conclusions:**

Serum cortisol levels significantly differed from normal values in methadone-treated patients. Therefore, we recommend measuring serum cortisol levels in methadone-treated patients before major medical procedures to consider the stress doses of corticosteroids.

## 1. Background

For thousands of years, narcotics have been used for medicinal and palliative purposes and still have an important role in relieving pain, diarrhea, cough, and other symptoms. Narcotics abuse has risen dramatically in recent years. For instance, Golestan Cohort Study conducted in Golestan province, Iran, reported that 17% (n = 8,487) of the participants' misused opium, with a mean duration of 12.7 years (1). Another study conducted in Fars province, Iran, reported that 8% (n = 339) of the participants misused opium (2). In the United States, 3% to 4% of adults receive long-term opioid treatment (3).

Along with opioid use and misuse, there are unfavorable side effects, including endocrinopathies due to long-time opioid usage (3). Endocrinopathy of narcotics should be considered for any patient using the equivalent of 100 mg of morphine per day or more. Measuring the response of plasma cortisol levels to intravenous or muscular injections of ACTH is a common screening test for detecting adrenal insufficiency. Various diagnostic criteria have been set according to base cortisol levels, stimulated cortisol levels, or their difference (4).

Methadone is a synthetic opioid. A complete Mu (µ) receptor agonist may mimic endogenous opioids, enkephalins, and endorphins (5). Methadone is frequently used to relieve pain, especially in the intensive care unit (ICU), and ease quitting opium addiction. This drug is qualitatively equivalent to morphine but has a longer half-life. The plasma half-life of methadone is very long and variable (13 - 100 hours). Despite this feature, many patients need methadone every 4 - 8 hours to maintain the analgesic effects (6).

Methadone maintenance therapy (MMT) has shown excellent results in managing heroin-dependent patients. However, the researchers questioned whether MMT could improve the function of the hypothalamic-pituitary-adrenal (HPA) axis, which is damaged by heroin dependence, and improve baseline cortisol levels. In this regard, studies are limited and contradictory. For instance, in a 2006 study by Aouizerate et al., methadone reduced serum cortisol levels. However, a 2016 study by Young et al. found increased body cortisol levels following methadone administration (6, 7).

Detecting adrenal insufficiency is critical, especially in ICU patients and patients undergoing major surgeries. To the best of our knowledge in the available research, adrenal insufficiency in opium-addicted patients on MMT has not been evaluated with a cosyntropin test (ACTH stimulation test) (8).

## 2. Objectives

Regarding the high prevalence of methadone use, we aimed to measure the changes in cortisol levels and the response to the cosyntropin test to determine the extent and prevalence of adrenal insufficiency in opium-addicted patients on methadone treatment.

## 3. Methods

This study was conducted in November 2019 at Imam Reza Hospital Rehab Center, Birjand, Iran. The patients were on methadone to ease quitting opium addiction. Our inclusion criteria were an addiction to opium for at least six months, not using corticosteroids in the past year, age of 20 to 45, not having significant co-morbidities such as diabetes or cancer, and no history of quitting opium addiction. According to a study by Annane et al. (9) which reported a mean cortisol level of 13.9 ± 10.3 in its population, a sample size of 42 was calculated with α = 0.01 and β = 0.1.

Convenience sampling was used to select patients. Eighty patients were assessed for eligibility, 42 of whom were enrolled in the study based on our inclusion criteria. The study procedure was explained to the patients; those who filled out informed consent and met the inclusion criteria were enrolled. A questionnaire was filled out to gather demographic characteristics. Cosyntropin tests were performed at 8-9 AM to minimize the effect of circadian rhythm on cortisol levels. Initially, a 5 mL blood sample was obtained to measure baseline cortisol. Afterward, 250 micrograms of intra-muscular cosyntropin were injected. In 30- and 60-minute intervals, blood samples were taken. The samples were analyzed at the central laboratory of Imam Reza Hospital. Chemiluminescence detection was used to measure cortisol levels with kits from Saluggia company, Italy.

According to Henry's Clinical Diagnosis and Management by Laboratory Methods (10), following the cosyntropin test, cortisol levels should be higher than 18 µg/dL, and lower levels determine adrenal insufficiency. Also, according to a study by Annane et al. (9), a cortisol level change of less than 9 µg/dL is considered adrenal insufficiency. We used these definitions of adrenal insufficiency in our study. Also, based on the chemiluminescence device's reference, which measured cortisol, the mean cortisol level in the standard population is 14 µg/dL (11). We compared our population with this value.

All statistical analyses were performed using SPSS version 16 software (SPSS Inc., Chicago, Illinois, USA). The normal distribution of variables was evaluated using the Kolmogorov–Smirnov test. Descriptive data are shown as the mean ± standard deviation or number (%). One-way analysis of variance (ANOVA) was applied to compare the demographic and clinical features between the groups. Repeated-measures ANOVA was recruited to assess the effect of cosyntropin on cortisol levels. Degrees of freedom were adjusted via Mauchly's W test, followed by a Greenhouse-Geisser correction of P-values.

## 4. Results

As shown in Table 1, the mean age of the participants was 34.4 ± 5.2, and most were men (90.5%). Eight of them were cigarette smokers.

**Table 1.** Demographic and Clinical Characteristics of Study Participants ^a^

Variables	Values
**Age **	34.4 ± 5.2
**BMI **	22.5 ± 3.6
**Gender**	
Male	38 (90.5)
Female	4 (9.5)
**Smoking**	8 (19)
**Corticosteroid use**	1 (2.4)
**Comorbidity**	
Thyroid disease	1 (2.4)
COPD	1 (2.4)

^a^ Values are expressed as Mean ± SD or No. (%).

Cortisol levels and response to the cosyntropin test had a normal distribution (P-value = 0.44 and 0.28, respectively).

The mean serum cortisol level at baseline was 9.46 ± 5.42 µg/dL, significantly different from its normal value of 14 µg/dL (P < 0.001). The mean response to the cosyntropin test (difference from baseline) was 9.34 ± 8.11 µg/dL.

According to Henry's Clinical Diagnosis and Management by Laboratory Methods (10), 21 (50.0%) participants had adrenal insufficiency, and according to the study of Annane et al. (9), 24 (57.1%) participants had adrenal insufficiency.

**Figure 1.**
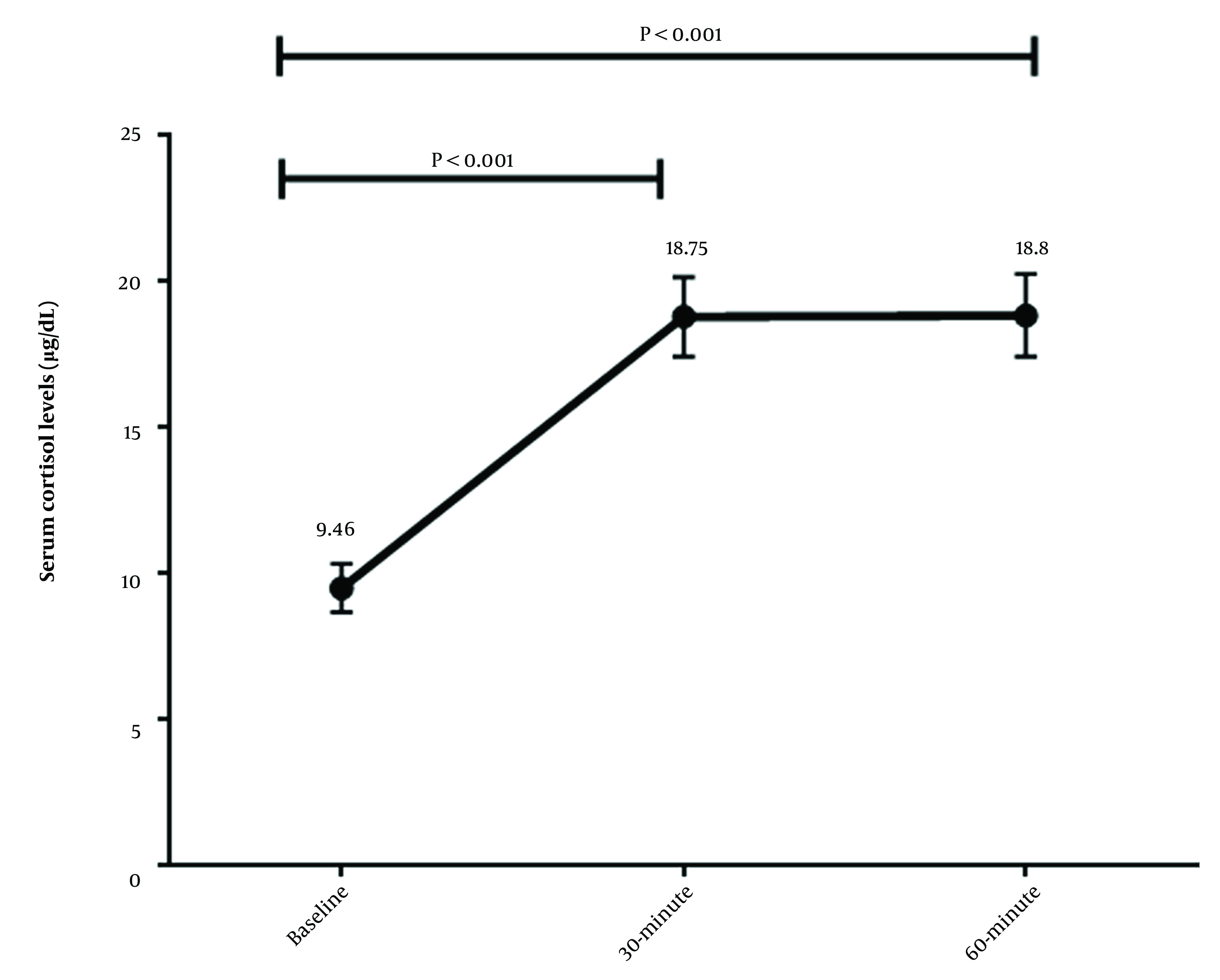
Changes in serum cortisol level in response to cosyntropin test

There was a significant difference between baseline cortisol levels and cortisol levels at 30- and 60-minute intervals (P-values < 0.001) (Figure 1).

Mean baseline cortisol levels and response to cosyntropin were not associated with age, dose, and duration of methadone usage (Table 2).

**Table 2.** The Relationship Between Baseline Cortisol Levels and Response to Cosyntropin Test with Other Variables

Variables	No. (%)	Mean Baseline Cortisol (µg/dL)	P-Value ^a^	Response to Cosyntropin Test (µg/dL)	P-Value ^a^
**Duration of methadone usage, mo**			0.08		0.40
< 1	6 (14.3)	9.12 ± 2.75		5.75 ± 3.77	
1 – 6	9 (21.4)	12.96 ± 7.72		8.30 ± 6.56	
< 6	27 (64.3)	8.36 ± 4.60		10.48 ± 9.11	
**Dosage of methadone used**			0.72		0.89
< 100	16 (38.1)	9.07 ± 5.57		9.13 ± 9.85	
< 100	26 (61.9)	9.70 ± 5.43		9.47 ± 7.03	
**Age, y**			0.13		0.39
25 - 30	5 (11.9)	9.64 ± 3.42		6.00 ± 5.41	
31 - 35	7 (16.7)	5.39 ± 2.06		13.01 ± 10.59	
36 - 40	18 (42.9)	11.06 ± 6.43		8.01 ± 5.54	
41 - 45	12 (28.6)	9.35 ± 4.95		10.60 ± 10.36	

^a^ By using one-way ANOVA

## 5. Discussion

This study investigated changes in cortisol levels and the response to ACTH hormone in former opium addicts on methadone treatment. Considering adrenal insufficiency can affect the management of these patients. Fifty-five percent of our participants had cortisol levels lower than 18 µg/dL following the cosyntropin test, indicating adrenal insufficiency.

Many earlier studies show that chronic opioid misuse can lead to HPA axis suppression. In this regard, a review by Donegan and Bancos concluded that 9 to 29% of patients receiving long-term opiate therapy may experience opioid-induced adrenal insufficiency. However, our study's prevalence of adrenal insufficiency was significantly higher (3). Whether MMT restores the disrupted HPA axis is still unknown, and studies in this regard are inconsistent.

Some studies have shown an overactivated HPA axis in MMT patients compared to controls (12-14). A study by Yang et al. on 52 MMT patients and 41 age-matched controls showed that MMT patients had significantly higher hair cortisol levels than the controls. Likewise, MMT patients showed significantly higher perceived stress levels. The authors imply that this higher stress level may have masked the suppressed HPA axis (7). In contrast, in a study, heroin users showed normal HPA activation with metyrapone, an 11-beta-hydroxylase inhibitor. The same study showed that patients on MMT addicted to cocaine had a hyperactivated HPA response to metyrapone (15). Dackis et al. studied five methadone misusers and 12 controls and observed a decreased response to ACTH stimulation in methadone misusers (16). Some case reports have also shown that chronic use of opioids can cause adrenal insufficiency (17, 18).

As mentioned, studies on cortisol levels and HPA axis function in patients on MMT are contradictory, and to date, the reasons for this discrepancy are unclear (19). One possible explanation may be that studies have used plasma, saliva, and urine cortisol levels as biological markers to assess basal cortisol levels. These biological markers are prone to circadian rhythms and events before sampling. Recently, endogenous cortisol levels in human hair have been proposed to overcome limitations and indicate cortisol over up to six months (7). Another explanation may be that participants in previous studies have been at different stages of the detoxification reaction. In addition, the activity of the HPA axis in patients on MMT may be affected by negative emotions. For example, patients with depressive symptoms may have higher basal cortisol levels. In addition, psychological and MMT factors may have synergistic effects on HPA axis function (20, 21). Differences in opioid receptor affinity due to polymorphisms in different individuals may be another explanation (22). The duration of MMT can affect the result of studies. Kreek et al. showed that metyrapone and ACTH stimulation tests were abnormal in the first two months of MMT but normal after two months (23). In line with this, response to the cosyntropin test was increased in longer MMT durations in our study (Table 2); however, this finding was not statistically significant (P-value = 0.40). The mechanism of HPA axis normalization is not clear. One explanation is given by Kling et al., who used positron emission tomography (PET) to study opiate receptors in MMT patients. They observed that only 19 – 32% of opiate receptors were occupied, and the remaining receptors could function normally in the HPA axis (24).

Adrenal insufficiency can cause hemodynamic disturbances, changes in consciousness, hypoxemia, and ileus. It can be life-threatening if not managed properly (25). However, most of the patients have non-specific symptoms that may mislead clinicians. Therefore, knowing that many opioid abusers and MMT patients may suffer from adrenal insufficiency can help prevent serious complications in case of major medical stress. Cortisol helps maintain the balance of the cardiovascular system during surgical trauma by facilitating the activity of catecholamines. In this regard, Baghaei Wadji et al. examined the effects of opium addiction on the response to the stress of major surgeries. The serum cortisol level of the addict group showed a significant increase compared to the non-addict group 24 hours after surgery, indicating a stronger response of opium addicts to surgical stress (26). Cortisol levels during and after surgery are proportional to the severity of the operation, and any disturbances, whether an inappropriate increase like in the mentioned study or an inappropriate decrease like in our study, can be life-threatening (27).

Most studies confirm that opioid misuse suppresses the HPA axis. However, whether long-term MMT can normalize the HPA axis is still unknown. Larger studies with control groups are needed to answer this question.
